# Diagnostic and therapeutic management of a left forearm tumor in a violinist: Clinical case and literature review^☆^

**DOI:** 10.1016/j.ijscr.2025.110944

**Published:** 2025-01-30

**Authors:** Thomas Daoulas, Flore-Anne Lecoq

**Affiliations:** Institut Main Nantes Atlantique, Bd Charles-Gautier, 44800 Saint-Herblain, France

**Keywords:** Case report, Dystonia, Musician

## Abstract

**Introduction:**

The management of forearm tumors requires a precise clinical examination and the performance of further examinations before considering possible surgical management is necessary, particularly in the case of professional musicians. In this specific population, certain differential diagnoses may fail to identify an organic cause for a painful symptomatology. Among the known causes is musician's focal dystonia (https://pubmed.ncbi.nlm.nih.gov/20590806/, n.d.; Rozanski et al., 2015).

**Case:**

65-year-old patient, professional violinist, who consulted for a tumor of the forearm, with involuntary tremor-like movements which recently made it impossible to play the violin. Tumor removal was performed and anatomopathological analysis revealed a schwannoma. The patient was able to play the violin from the first week after surgery and the tremors disappeared.

**Discussion:**

Musician's dystonia could be envisaged here, given the presence of discomfort only during instrumental practice. The management of this tumor required a precise clinical examination, with the musician's instrument. Musician's dystonia must remain a diagnosis of elimination.

**Conclusion:**

We report a misleading case of a nerve tumor in a musician that could easily be mistaken for dystonia at first glance.

## Highlights

• Musicians have specific fuctional needs.
• Some cystic lesions can mimic focal dystonia.
• Certain non-physiological movements may be compromised by the presence of a lesion, even though it does not interfere with everyday movements.
• A forearm schwannoma caused parasitic 4th/5th finger movements, interfering with violon playing.

## 1 Introduction

The management of forearm tumors requires a precise clinical examination and the performance of further examinations before considering possible surgical management is necessary, particularly in the case of professional musicians.

## 2 Presentation of case

We report here the case of a 65-year-old violinist referred by his general practitioner to a hand specialist for the following reason: “cyst of the left forearm associated with hand tremors”.

## 3 Interrogatory

The patient is a 65-year-old professional violinist and choirmaster, recently retired from the public service and has been playing the violin since the age of 6. At his usual rhythm, he plays the violin more than 4 h a day. He has no notable antecedents apart from active smoking (45 pack-years). In particular, he has no family history of diabetes, dysthyroidism or tremor.

The tumor is located at the junction between the middle 1/3 and distal 1/3 of the forearm, on the palmar surface. It has existed for many years (over 15) and was previously asymptomatic. However, according to the patient, it has progressively increased in volume over the past few months (currently around 2 cm long). Functional discomfort is only described when playing an instrument, and corresponds to the appearance of uncontrolled movements of the ring and little fingers, such as trembling and curling in flexion. The parasitic movement appears as soon as the instrument is played, with no free interval.

It is accompanied by a loss of note accuracy and precision of musical phrasing. There is no spontaneous pain, particularly at the onset of uncontrolled movement. On a day-to-day basis, there is no functional discomfort: the patient tells us that he would never have consulted us if he had no discomfort with his instrumental playing. The symptoms appeared 6 months ago and rapidly intensified, making it impossible for him to play the violin, and he has not played his instrument for 4 months. At his last concert before quitting, he involuntarily performed left-hand pizzicati while playing Bach's Brandenburg Concerto, and was unable to finish the piece. No further examination was ever carried out to explore the lesion.

Written informed consent was obtained from the patient for publication and any accompanying images. A copy of the written consent is available for review by the Editor-in-Chief of this journal on request.

## 4 Clinical examination

Clinical examination revealed a tumor on the anterior aspect of the left forearm at the mid-third-distal junction, with no reaction from the soft tissues, mobile but indurated. There is a painful Tinel on percussion, but no pain on palpation. There is no hypoesthesia or motor deficit.

When the forearm is placed in maximum supination, in a position mimicking that of holding the neck of a violin, the parasitic movement is reproduced (Video 1). On examination with his instrument, the parasitic movement is present during the consultation (Video 2). There is a lack of fingerboard coverage when he plays, a loss of strength and fatigability.

Did this movement correspond to focal musician's dystonia, or was it really linked to this tumor? The arguments for and against these two diagnoses are reported in Table 1 at this stage of the diagnostic process [1,2].

**Table 1:** Arguments for focal musician's dystonia or an organic cause on clinical examination.

	Arguments in favor of dystonia	Arguments for an organic cause
Patient	Professional musician	Age
	Intensive daily practice for many years	
Location	A violinist's left hand	On the tumor side
Symptoms	Uncontrolled movement	Tinel positive
	Pain-free	
Chronology	Lesion present 15 years before onset of clinical signs	Recent volume increase
Circumstances of onset	Hindrance only to violin playing, from the very beginning of practice	Movement reproduced when instrument position is simulated

## 5 Paraclinical examinations

Ultrasound examination revealed an oval tissue mass measuring 8 × 16 × 19 mm, with circumscribed contours, hypoechoic with no posterior enhancement, and peripheral vascularization. It had a mass effect on the flexor tendons of the ring and little fingers, without invading the latter.

**Fig. 1:**
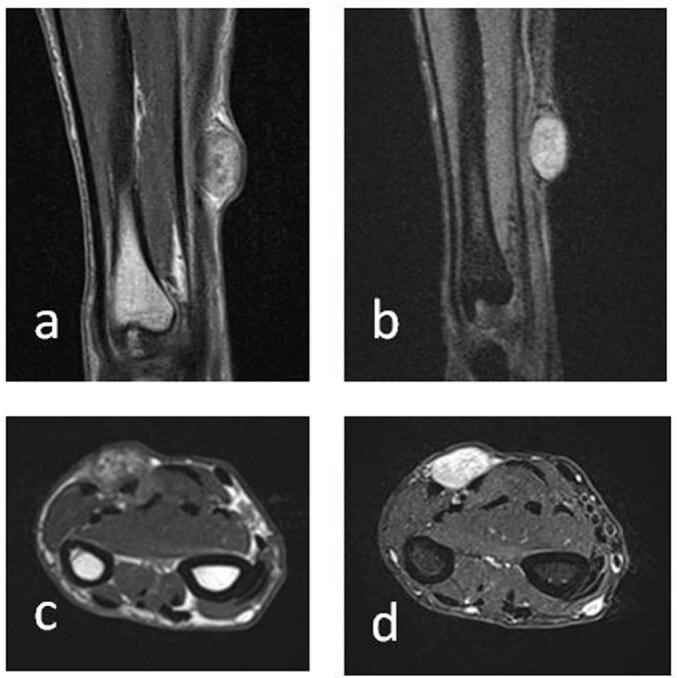
MRI of the forearm showing the lesion. A) T2-weighted coronal section B) Gadolinium-enhanced T1-weighted coronal section C) T2-weighted axial section D) Gadolinium-enhanced T1-weighted axial section.

MRI revealed a subcutaneous tissue swelling measuring 19 × 16 × 10 mm, located on the immediate surface of the antebrachial fascia. The lesion showed a frank, heterogeneous T2 hypersignal, moderately enhanced after injection (Fig. 1). It exerts a mass effect on the underlying muscles, with edema opposite the flexor digitorum superficialis of the long fingers.

An electromyogram and a specialist opinion from a neurologist were requested. The sensory and motor potentials of the ulnar, median and radial sensory nerves were normal, and there was no argument for a central origin. The neurologist did not recommend any additional central morphological exploration.

Complementary examinations thus supported an organic rather than a dystonic origin for the patient's symptoms. Surgery was proposed to remove the supraaponeurotic lesion under locoregional anaesthesia on an outpatient basis, with anatomopathology analysis.

**Fig. 2:**
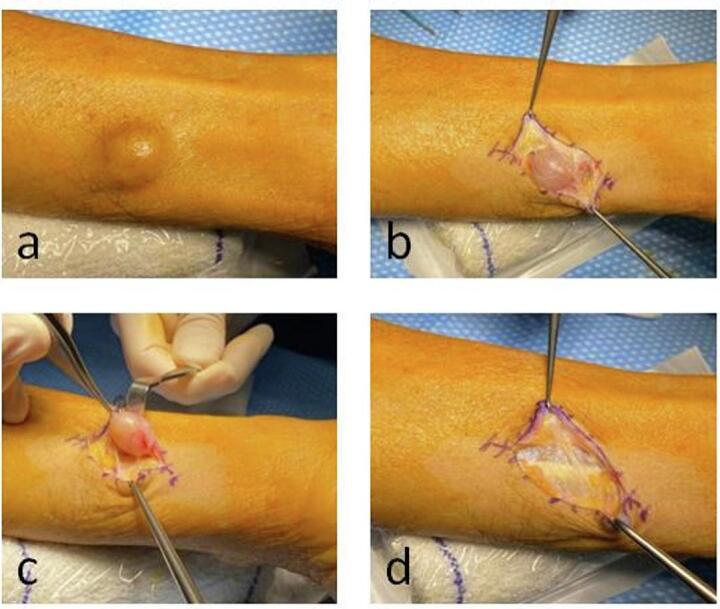
Intraoperative photographs of the lesion. A) Subcutaneous appearance of the lesion. B) Dissection and demonstration of a well-defined cystic lesion. C) Macroscopically complete removal of the lesion. D) Surgical zone after lesion removal.

The incision was centered over the swelling. Intraoperatively, under magnifying glasses, an encapsulated cystic lesion was found, supraaponeurotic and non-adherent in depth (Fig. 2). The lesion was in contact with the fascia of the flexor carpi ulnaris, flexor digitorum superficialis of the 5th finger, and flexor digitorum profundus of the 5th finger. However, the lesion did not penetrate the fascia and remained well encapsulated. The ulnar nerve was neurolyzed along the entire length of the incision, with the superficial branch of the ulnar nerve located distally to the operative field. Given the presence of a well-encapsulated tumor, the dissection was not extended into deeper tissues, specifically avoiding penetration of the fascia of the flexor muscles. The tumor was successfully excised en bloc without compromising the continuity of the ulnar nerve. Anatomopathology analysis was performed.

## 6 Post-operative care

**Fig. 3:**
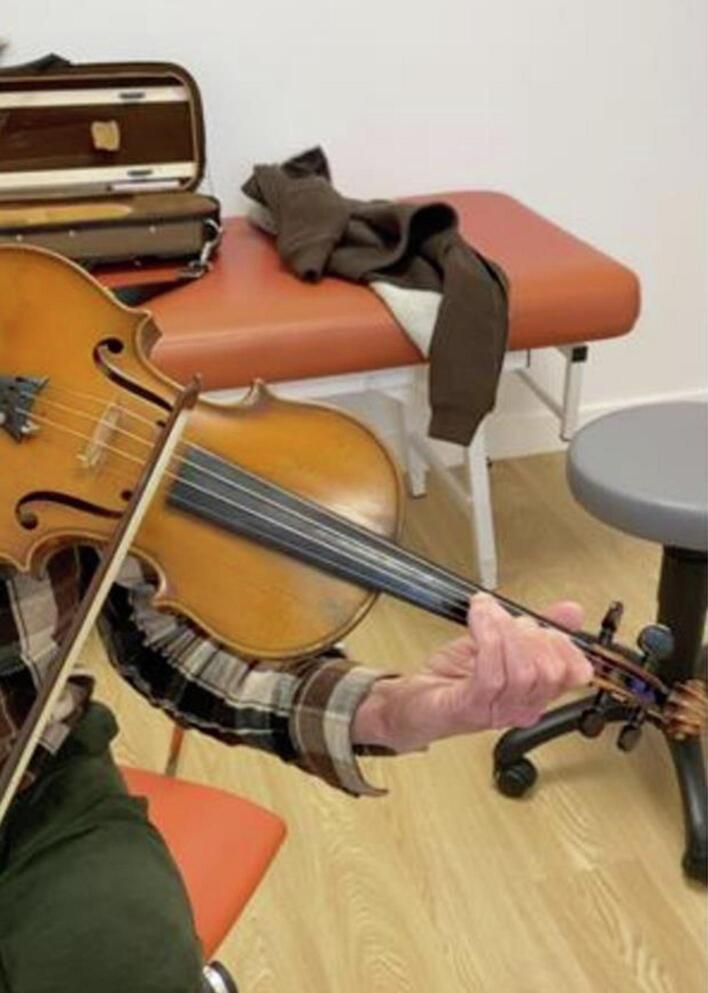
Clinical examination with violin at 1 month post-operatively. Disappearance of symptoms bothering the patient.

The patient was able to start again violin playing one week after surgery and quickly felt a functional improvement in both involuntary movements and strength. Resumption was gradual, but still with daily practice, accompanied by advice on stretching and softening the left upper limb. He was reviewed in consultation at 1 month (Fig. 3), 6 months, 1 and 2 years.

With a 2 years follow-up, the scar was not adherent, there was no allodynia, no downstream hypoesthesia, and the pain VAS was 0 at rest and during music practice. The preoperative Tinel had disappeared. He was now able to cover the fingerboard with the ulnar rays without difficulty. The tremors disappeared progressively and were negligible at 6 months post-operatively. They had completely disappeared by the last follow-up. The patient was very satisfied with the result at the last follow-up (2 years).

Anatomopathological analysis revealed a slightly reshaped schwannoma me, measuring 2 × 1.4 × 1 cm, completely excised, with no sign of malignancy. The schwannoma had in fact probably developed at the expense of a small cutaneous sensory branch of the ulnar nerve, which is why it was not possible to individualize it on MRI.

## 7 Discussion

Schwannomas, which develop at the expense of Schwann cells, are the most common benign tumors of the peripheral nervous system [3]. They are well-encapsulated tumors of slow progression [4]. They often involve mixed nerves and account for less than 3 % of benign soft-tissue tumors of the upper limb [5], mostly on the palmar surface. In the upper limb, the nerve most frequently affected is the ulnar nerve [6], but median and radial nerves, as well as sensory branches and collateral nerves, may also be involved. It is not always possible to identify the affected nerve pre-operatively, as was the case with this patient.

It is most often a single tumor, 1 to 3 cm in diameter [7]. It predominantly affects middle-aged adults (mean age at diagnosis 45 years), with no difference in prevalence according to gender [8]. In the literature, clinical cases are often described, and there are few large-volume series [[6], [7], [8], [9]]. Clinical and radiological outcomes may be similar to those of other soft tissue tumors. Tinel's sign is present in over 80 % of patients [6].

Treatment involves one-piece surgical excision with microsurgical dissection and initial biopsy is rarely required [7]. Anatomopathological analysis with immunohistochemical study confirms the diagnosis with certainty [10]. After surgical management, functional recovery is generally good. Nevertheless, complications are not uncommon, and Knight [6] reports persistent pain in 30 % of patients, hypoesthesia in 20 % and loss of strength in 12 %. These complications have a particularly significant impact on musicians with high functional demands.

After appropriate surgical management, recurrence is rare, and no malignant transformation has been described to date [6,7].

In this patient, a professional musician, the clinical picture could initially point towards focal musician's dystonia, notably the fact that functional discomfort was present only during instrumental practice and the absence of pain. He had risk factors for dystonia [1,2]: long musical career, left hand in a violinist, classical music in an orchestra, long working week. Before making this diagnosis, it is necessary to carry out further tests to eliminate another cause [11].

An MRI appeared particularly indicated to characterize the tumor before surgery and rule out signs of invasion into the adjacent soft tissues [12].

The causal link between the lesion and the symptoms was not immediately obvious. The pathophysiology was essentially explained by the mass syndrome exerted by the lesion on the flexor tendons at the myotendinous junction. The tremors induced by the tumor are likely secondary to its myotendinous location, which houses numerous mechanoreceptors [13,14]. This existed only in the position of maximum supination of the forearm. This remained asymptomatic as long as the lesion was not large enough to cause significant congestion. It is possible that some of the symptoms could also be explained by the nature of the lesion itself, being a nerve tumor. We hope that the presentation of this case will provide practitioners with an additional perspective when considering potential differential diagnoses for musician's focal dystonia.

The work has been reported in line with the SCARE criteria [15].

## 8 Conclusion

The management of a forearm tumor, particularly in the case of a professional musician, calls for a rigorous diagnostic approach. It begins, of course, with a thorough history and clinical examination, with and without the instrument, which should be repeated at each consultation. The physician must refrain from hasty judgments and preconceptions, and take the necessary distance to fully assess the situation. Imaging must be performed before considering surgical management. Last but not least, clear and appropriate information is essential to ensure that the patient adheres to the treatment plan. Several consultations are generally necessary before planning a surgical procedure as serenely as possible when taking care of a professional musicist.

We hope that the description of this unusual case will help guide the hand surgeon when faced with atypical musician's dystonia.

The following are the supplementary data related to this article.

Video 1 Reproduction of the parasitic movement in forearm supination during violin practice. Video 1

Video 2 Pre-operative violin examination. Video 2

## Author contribution

FAL: Conceptualization; Data curation; Project administration; Resources.

TD: Writing; Formal analysis; Funding acquisition; Investigation; Methodology.

## Consent

Written informed consent was obtained from the patient for publication and any accompanying images. A copy of the written consent is available for review by the Editor-in-Chief of this journal on request.

## Ethical approval

Ethical approval for this study was provided by the Ethical Committee of the Institut de la main Santé Atlantique, France on 17 February 2022.

## Guarantor

Thomas DAOULAS.

## Research registration number

Not applicable.

## Declaration of Generative AI and AI-assisted technologies in the writing process

All authors certify that they have not used AI in the writing, data collection and design of this article.

## Funding

None.

## Conflict of interest statement

The authors declare no conflict of interest.
